# Association of the transthyretin variant V122I with polyneuropathy among individuals of African ancestry

**DOI:** 10.1038/s41598-021-91113-6

**Published:** 2021-06-02

**Authors:** Margaret M. Parker, Scott M. Damrauer, Catherine Tcheandjieu, David Erbe, Emre Aldinc, Philip N. Hawkins, Julian D. Gillmore, Leland E. Hull, Julie A. Lynch, Jacob Joseph, Simina Ticau, Alexander O. Flynn-Carroll, Aimee M. Deaton, Lucas D. Ward, Themistocles L. Assimes, Philip S. Tsao, Kyong-Mi Chang, Daniel J. Rader, Kevin Fitzgerald, Akshay K. Vaishnaw, Gregory Hinkle, Paul Nioi

**Affiliations:** 1 Alnylam Pharmaceuticals, 300 3rd St., Cambridge, MA 02142 USA; 2 Department of Surgery, Perelman School of Medicine, University of Pennsylvania, Philadelphia, PA 19104 USA; 3 The Corporal Michael J. Crescenz VA Medical Center, Philadelphia, PA 19104 USA; 4 12 VA Palo Alto Health Care System, Palo Alto, CA 94304 USA; 5 Department of Medicine, Stanford University School of Medicine, Stanford, CA 94304 USA; 6 Centre for Amyloidosis & Acute Phase Proteins, Division of Medicine UCL (Royal Free Campus), London, NW3 2PF UK; 7 Division of General Internal Medicine, Massachusetts General Hospital, Boston, MA 02114 USA; 8 Center for Healthcare Organization and Implementation Research, Edith Nourse Rogers Memorial Veterans Hospital, Bedford, MA 01730 USA; 9 School of Nursing & Health Sciences, University of Massachusetts, Boston, MA 02125 USA; 10 VA Informatics and Computing Infrastructure (VINCI), VA Salt Lake City Health Care System, Salt Lake City, UT 84148 USA; 11 Department of Medicine, Veterans Affairs Boston Healthcare System, Boston, MA 02130 USA; 12 Department of Medicine, Brigham and Women’s Hospital and Harvard Medical School, Boston, MA 02115 USA; 13 Department of Medicine, Perelman School of Medicine, University of Pennsylvania, Philadelphia, PA 19104 USA; 14 Department of Genetics, Perelman School of Medicine, University of Pennsylvania, Philadelphia, PA 19104 USA

**Keywords:** Computational biology and bioinformatics, Drug discovery, Genetics, Physiology, Diseases, Signs and symptoms

## Abstract

Hereditary transthyretin-mediated (hATTR) amyloidosis is an underdiagnosed, progressively debilitating disease caused by mutations in the transthyretin (*TTR*) gene. V122I, a common pathogenic *TTR* mutation, is found in 3–4% of individuals of African ancestry in the United States and has been associated with cardiomyopathy and heart failure. To better understand the phenotypic consequences of carrying V122I, we conducted a phenome-wide association study scanning 427 ICD diagnosis codes in UK Biobank participants of African ancestry (*n* = 6062). Significant associations were tested for replication in the Penn Medicine Biobank (*n* = 5737) and the Million Veteran Program (*n* = 82,382). V122I was significantly associated with polyneuropathy in the UK Biobank (odds ratio [OR] = 6.4, 95% confidence interval [CI] 2.6–15.6, *p* = 4.2 × 10^−5^), which was replicated in the Penn Medicine Biobank (OR = 1.6, 95% CI 1.2–2.4, *p* = 6.0 × 10^–3^) and Million Veteran Program (OR = 1.5, 95% CI 1.2–1.8, *p* = 1.8 × 10^−4^). Polyneuropathy prevalence among V122I carriers was 2.1%, 9.0%, and 4.8% in the UK Biobank, Penn Medicine Biobank, and Million Veteran Program, respectively. The cumulative incidence of common hATTR amyloidosis manifestations (carpal tunnel syndrome, polyneuropathy, cardiomyopathy, heart failure) was significantly enriched in V122I carriers compared with non-carriers (HR = 2.8, 95% CI 1.7–4.5, *p* = 2.6 × 10^−5^) in the UK Biobank, with 37.4% of V122I carriers having at least one of these manifestations by age 75. Our findings show that V122I carriers are at increased risk of polyneuropathy. These results also emphasize the underdiagnosis of disease in V122I carriers with a significant proportion of subjects showing phenotypic changes consistent with hATTR amyloidosis. Greater understanding of the manifestations associated with V122I is critical for earlier diagnosis and treatment.

## Introduction

Hereditary transthyretin-mediated (hATTR) amyloidosis, also known as ATTRv (v for variant) amyloidosis, is an underdiagnosed, progressively debilitating, and fatal disease caused by mutations in the transthyretin (*TTR*) gene. These pathogenic variants result in misfolding of TTR proteins, which accumulate as amyloid deposits in multiple tissues throughout the body, including the heart, nerves, and gastrointestinal tract^1–3^. Organ involvement and symptoms of hATTR amyloidosis can vary by patient and mutation, but carriers of pathogenic variants have an increased risk of developing life-impacting polyneuropathy, cardiomyopathy, and other symptoms (e.g., carpal tunnel syndrome [CTS])^4,5^.

One of the most common pathogenic *TTR* variants is the valine-to-isoleucine substitution at position 122 (V122I; p.V142I; rs76992529), which is predominantly found in individuals of West African descent^6^, with 3–4% of the African–American population carrying the mutation^7^. The variant was originally identified in cardiac TTR amyloid deposits^8^, and subsequent studies demonstrated it to be a common cause of heart failure (HF) among elderly African patients^9,10^. V122I hATTR amyloidosis is substantially underdiagnosed^5^, and patients are often at an advanced disease stage when finally diagnosed. This long journey to diagnosis also results in poor outcomes for these patients, with median time from diagnosis to death being 2.6 years^9^. Thus, a better understanding of early hATTR amyloidosis disease manifestations is critical to expedite diagnosis and initiate treatment^11^. Unlike some other common *TTR* mutations, the V122I variant has historically been associated with a predominantly cardiac phenotype. However, a better understanding of the full range of disease manifestations associated with this variant is critical to improve disease recognition and allow maximal benefit from currently available therapies.

The present study assessed the association of the V122I genotype with ICD10 (International Statistical Classification of Diseases and Related Health Problems, 10th Revision) disease diagnoses in the UK Biobank, a large, population-based, prospective cohort study^12^. The UK Biobank study does not recruit participants based on any disease outcome, thus affording the unique opportunity to analyze the V122I variant in a population not influenced by referral bias, allowing better understanding of early disease manifestations and variant penetrance. Significant associations were assessed for replication in the Penn Medicine Biobank and Million Veteran Program.

## Methods

### Study design

This phenome-wide association study and Cox regression of V122I carriers and non-carriers assessed the association between the V122I variant and ICD diagnoses, including common manifestations of hATTR amyloidosis, in three large biobanks (UK Biobank, Penn Medicine Biobank, and Million Veteran Program). Written informed consent was obtained from all study participants and all methods were performed in accordance with the relevant guidelines and regulations**.**

### Study population

The UK Biobank recruited ~ 500,000 participants in England, Wales, and Scotland between 2006 and 2010^13^. The Penn Medicine Biobank includes over 60,000 participants over age 18 years enrolled through the University of Pennsylvania Health System since 2008. The Million Veteran Program is a large, multiethnic cohort within the US Department of Veterans Affairs which includes more than 825,000 veterans over age 18 years who have been recruited since 2011 across the United States. Additional details on the biobanks used in this study are provided in the “Supplementary Information”.

This study included 6062 unrelated African ancestry participants of the UK Biobank, defined based on a combination of self-reported ancestry and genetic principal components. To define the African ancestry population, we started with participants of self-reported African or Caribbean ancestry. Using genetic principal components one to six provided by the UK Biobank, we ran the R package aberrant^14^ (lambda value = 40) to identify and exclude population outliers^15^. An additional principal component analysis on the remaining African ancestry subset using PLINK v.2.0^16^ generated the principal components used as covariates to control for population stratification. First-and second-degree relatives (defined based on genetics) were removed from analysis.

The Penn Medicine Biobank analysis utilizes data on 5737 individuals of genetically inferred African ancestry that passed quality control as described in detail elsewhere^5^. Additionally, genotyped and imputed genetic information was available for 468,961 multiethnic participants from the Million Veteran Program, among which 87,163 participants (including related individuals) were of HARE-assigned African ancestry^17^. Of these, 82,382 unrelated participants of African ancestry were included in the current study^18^.

### V122I genotyping

The V122I genotype was directly typed by the UK Biobank on either the UK BiLEVE or the UK Biobank Axiom arrays^19^, which share > 95% common content. Genotypic data were imputed to the UK10K haplotype, 1,000 Genomes phase III, and Haplotype Reference Consortium reference panels using SHAPEIT3^20^ for phasing and IMPUTE4^15^ for imputation. The V122I variant was directly genotyped on both arrays and passed all quality control metrics (Hardy–Weinberg equilibrium *p* = 7.2 × 10^–5^, genotyping call rate = 100%), as detailed by Bycroft et al.^21^. Patients with and without the V122I genotype are referred to as “carriers” and “non-carriers,” respectively. The presence of other *TTR* genotypes was not examined in these populations.

### Statistical analysis

Descriptive statistics are presented as means (standard deviations) for continuous variables and percentages for categoric variables. Continuous and categoric variables were compared between V122I carriers and non-carriers using a t-test and a Pearson’s chi-squared test, respectively. All analyses were conducted using complete cases (i.e., no missing data).

### Phenome-wide association analysis and replication

To identify diagnoses associated with the V122I variant, a phenome-wide association analysis (PHEWAS) was performed on any primary or secondary inpatient ICD10 diagnosis code observed in at least 15 African ancestry participants from the UK Biobank (*n* = 427 ICD10 codes). PHEWAS was performed in PLINK^16^ using logistic regression controlling for age, sex, and genetic ancestry via ten principal components. A Bonferroni-corrected *p* = 1.2 × 10^–4^ was considered statistically significant. Significant associations with ICD9 and/or ICD10 diagnoses were assessed for replication in the Penn Medicine Biobank and Million Veteran Program using logistic regression controlling for age, sex, and genetic ancestry. Additional methodologic details are provided in the “Supplementary Information”.

### Assessment of common hATTR amyloidosis manifestations in V122I carriers

A variable was created to indicate if UK Biobank participants had been diagnosed with at least one of four common hATTR amyloidosis manifestations, independent of whether the participants had a diagnosis of hATTR amyloidosis, using the following ICD10 codes: polyneuropathy (“G62”), CTS (“G560”), cardiomyopathy (“I42”), and HF (“I50” or “I098”). The association between the V122I genotype and a diagnosis of a common hATTR amyloidosis manifestation was tested using logistic regression and Cox proportional hazards regression controlling for age, sex, and genetic ancestry via ten principal components; Kaplan–Meier curves were used to estimate cumulative incidence of common hATTR amyloidosis manifestations by V122I genotype. In addition to testing the composite phenotype, we tested each common hATTR amyloidosis manifestation separately using Cox proportional hazards regression (controlling for age, sex, and genetic ancestry) and Kaplan–Meier curves. A Bonferroni-corrected *p* < 0.0125 was considered statistically significant. To test if the association between V122I and common hATTR amyloidosis manifestations differed by sex, we performed two analyses: (1) Cox regression assessing time to first common hATTR amyloidosis manifestation including an interaction term of genotype × sex; and (2) performing a sex-stratified analysis to time to first hATTR amyloidosis manifestation. Additional methodologic details are described in the “Supplementary Information”.

### Characteristics of V122I carriers with common hATTR amyloidosis manifestations

It remains unclear why some V122I carriers develop hATTR amyloidosis while others do not. To better understand the characteristics of V122I carriers with manifestations, we performed a multiple logistic regression comparing V122I carriers with at least one common hATTR amyloidosis manifestation with V122I carriers without manifestations on five variables: age at study recruitment, sex, body mass index, cigarette smoking status, and the first genetic principal component, which served as a proxy for genetic ancestry. A Bonferroni-corrected *p*-value of 0.01 was considered statistically significant.

### Population attributable risk of common hATTR amyloidosis manifestations to the V122I genotype

The population risk of common hATTR amyloidosis manifestations attributable to the V122I genotype was calculated by multiplying the fraction of all diagnoses in V122I carriers by the risk difference between carriers and non-carriers. Risk was estimated using the cumulative incidence of the manifestations at age 75 for V122I carriers and non-carriers (additional methodologic details are described in the “Supplementary Information”).

### Ethics approval

The UK Biobank study was approved by the National Health Service National Research Ethics Service (ref. 11/NW/0382) and all participants provided written informed consent to participate in the UK Biobank study. Information about ethics oversight in the UK Biobank can be found at https://www.ukbiobank.ac.uk/ethics/. This research has been conducted using the UK Biobank resource, application no. 26041. The Penn Medicine Biobank research was approved by the Institutional Review Boards of the University of Pennsylvania (Protocol nos.: 808346 [CGI], 813913 [PMBB], and 817977 [PMBB tissue]). The Million Veterans Program research was approved by the Institutional Review Boards of the Veterans Affairs (Protocol no. 19-06—Genetics of Cardiometabolic Diseased in the VA Population II).

### Consent to participate

All necessary patient/participant consent has been obtained and the appropriate institutional forms have been archived.

## Results

### Baseline characteristics

A total of 387 of 487,327 genotyped UK Biobank participants carried the V122I variant, of which 80.6% were of self-reported African descent (see Fig. S1 in the “Supplementary Information”). Analyses were performed on a subset of these carriers that were unrelated and of genetically defined African ancestry. This unrelated African ancestry population totaled 6062 individuals, which included 240 heterozygotes and three homozygotes for the V122I variant (minor allele frequency = 2.0% or 1 in 50). Characteristics of this population are listed in Table 1. On average, participants were followed up for 7.6 years (range: 0.22–10.0 years) and were 59.6 years old at censoring (range: 42.2–79.0 years) (see Fig. S2 in the “Supplementary Information”). Baseline characteristics of V122I carriers and non-carriers from the Penn Medicine Biobank and Million Veteran Program are presented in Tables S1 and S2 in the “Supplementary Information”.

**Table 1:** Baseline characteristics of the African ancestry UK Biobank study population (*n* = 6062) by V122I genotype.

	Non-carriers (GG) (*n* = 5819)	Carriers (GA or AA) (*n* = 243)	*p*
Mean (SD) age at enrollment (years)	51.9 (8.1)	52.6 (8.2)	0.22
Male (%)	42.7	46.5	0.27
Mean (SD) BMI (kg/m^2^)	29.6 (5.4)	29.7 (5.1)	0.76
Hypertension (%)	36.8	39.7	0.39
Diabetes (%)	11.3	12.1	0.95
Smoking (%)	27.8	27.2	0.99

Among the 243 V122I carriers in the UK Biobank, 0.8% (*n* = 2) were formally diagnosed with amyloidosis (ICD10 diagnosis “E85”) compared with 0.1% (*n* = 5) of non-carriers. Both diagnosed patients were male heterozygous carriers, diagnosed at ages 75.9 and 71.9 years, and with cardiac manifestations of disease diagnosed during study follow-up. One of the two subjects died during study follow-up, with the primary cause of death being organ-limited amyloidosis (ICD10 diagnosis “E854”).

### Phenome-wide association analysis

**Figure 1:**
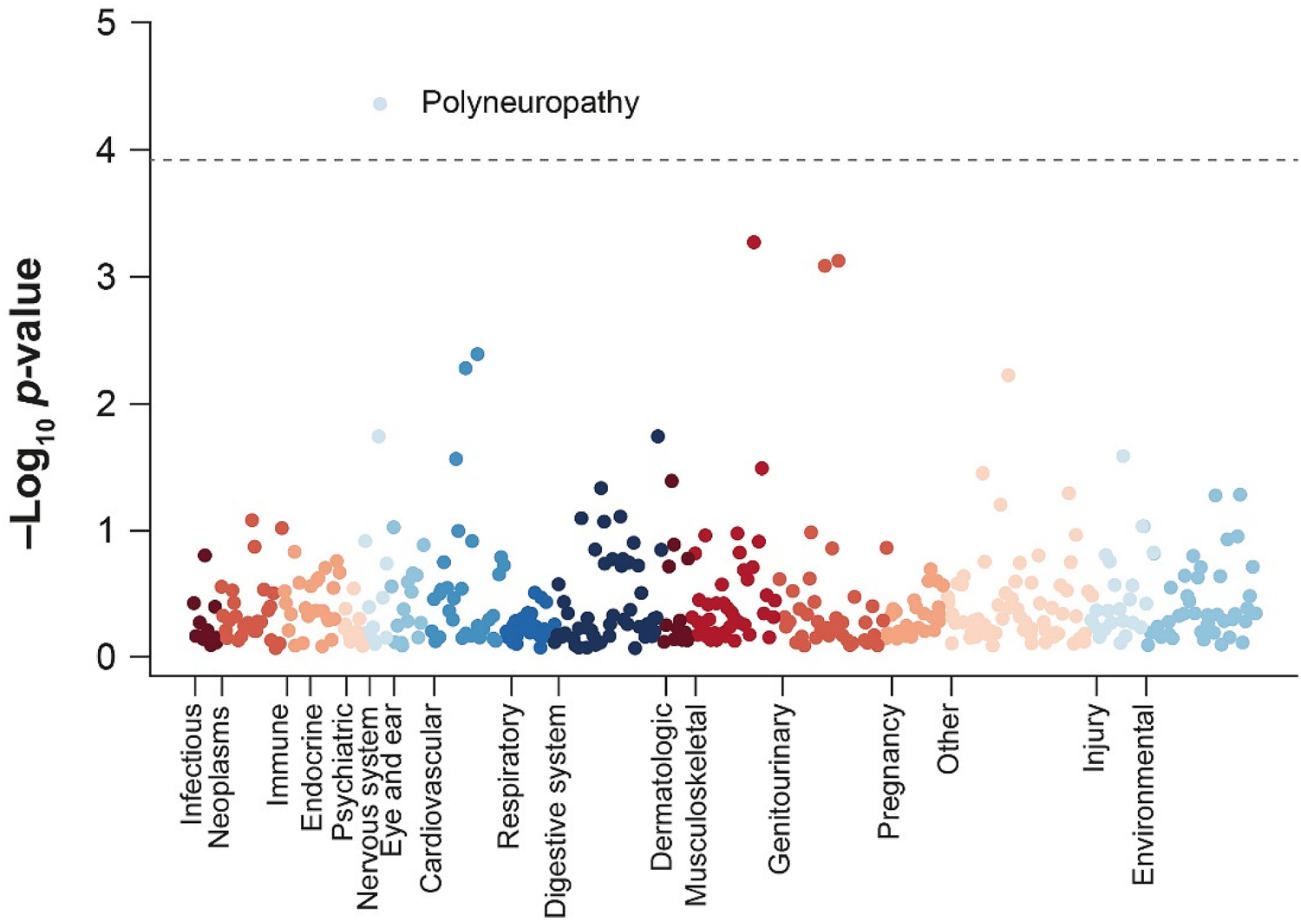
Phenome-wide association study of the V122I variant and 427 ICD10 diagnosis codes in the African ancestry UK Biobank population. The dotted gray line indicates a multiple comparison-corrected significance cutoff of *p* < 1.2 × 10^–4^. *ICD10* International Statistical Classification of Diseases and Related Health Problems, 10th Revision; *V122I* valine-to-isoleucine substitution at position 122.

To identify diagnoses associated with carrying the V122I variant, we performed PHEWAS that included 427 ICD10 diagnosis codes in the unrelated African ancestry UK Biobank subpopulation. After controlling for multiple comparisons, one diagnosis, polyneuropathy, was significantly associated with the V122I genotype (odds ratio [OR] = 6.4, 95% confidence interval [CI] 2.6–15.6, *p* = 4.2 × 10^–5^) (Fig. 1; Table S3 in the “Supplementary Information”). Significant associations between the V122I variant and polyneuropathy diagnosis were also seen in analyses of 190 V122I carriers and 5,547 non-carriers from the African ancestry population of the Penn Medicine Biobank (OR = 1.6, 95% CI 1.2–2.4, *p* = 6.0 × 10^–3^) and 2305 V122I carriers and 80,031 non-carriers from the African ancestry population of the Million Veteran Program (OR = 1.5, 95% CI 1.2–1.8, *p* = 1.8 × 10^–4^). In total, 2.1% (*n* = 5) of V122I carriers in the UK Biobank, 9.0% (*n* = 17) of V122I carriers in the Penn Medicine Biobank, and 4.8% (*n* = 111) of V122I carriers in the Million Veteran Program had a polyneuropathy diagnosis. The association of the V122I variant with polyneuropathy remained significant after adjustment for diabetes diagnosis (OR UK Biobank = 6.3, *p* = 6.4 × 10^–5^; OR Penn Medicine Biobank = 2.2, *p* = 4.6 × 10^–3^; OR Million Veteran Program = 1.5, *p* = 1.1 × 10^–4^).

### The V122I genotype and risk of common hATTR amyloidosis manifestations in the UK Biobank

In addition to phenome-wide analysis, we aimed to better understand the association between the V122I genotype and diagnosis of four common hATTR amyloidosis manifestations: polyneuropathy, CTS, cardiomyopathy, and HF, hereafter termed “common hATTR amyloidosis manifestations”. V122I carriers were significantly more likely to have at least one of these common diagnoses, with 11.1% of V122I carriers having at least one common hATTR amyloidosis manifestation compared with 4.9% of non-carriers (OR = 2.66, *p* = 1.5 × 10^–6^). Two of the three participants (66.7%) homozygous for V122I were diagnosed with at least one of the common hATTR amyloidosis manifestations, which was significantly greater than the proportion of heterozygous carriers (10.4%) diagnosed with at least one manifestation (*p* = 0.01). Significant associations between the V122I genotype and common hATTR amyloidosis manifestations were also observed in the Million Veteran Program (see Table S4 in the “Supplementary Information”).

**Figure 2:**
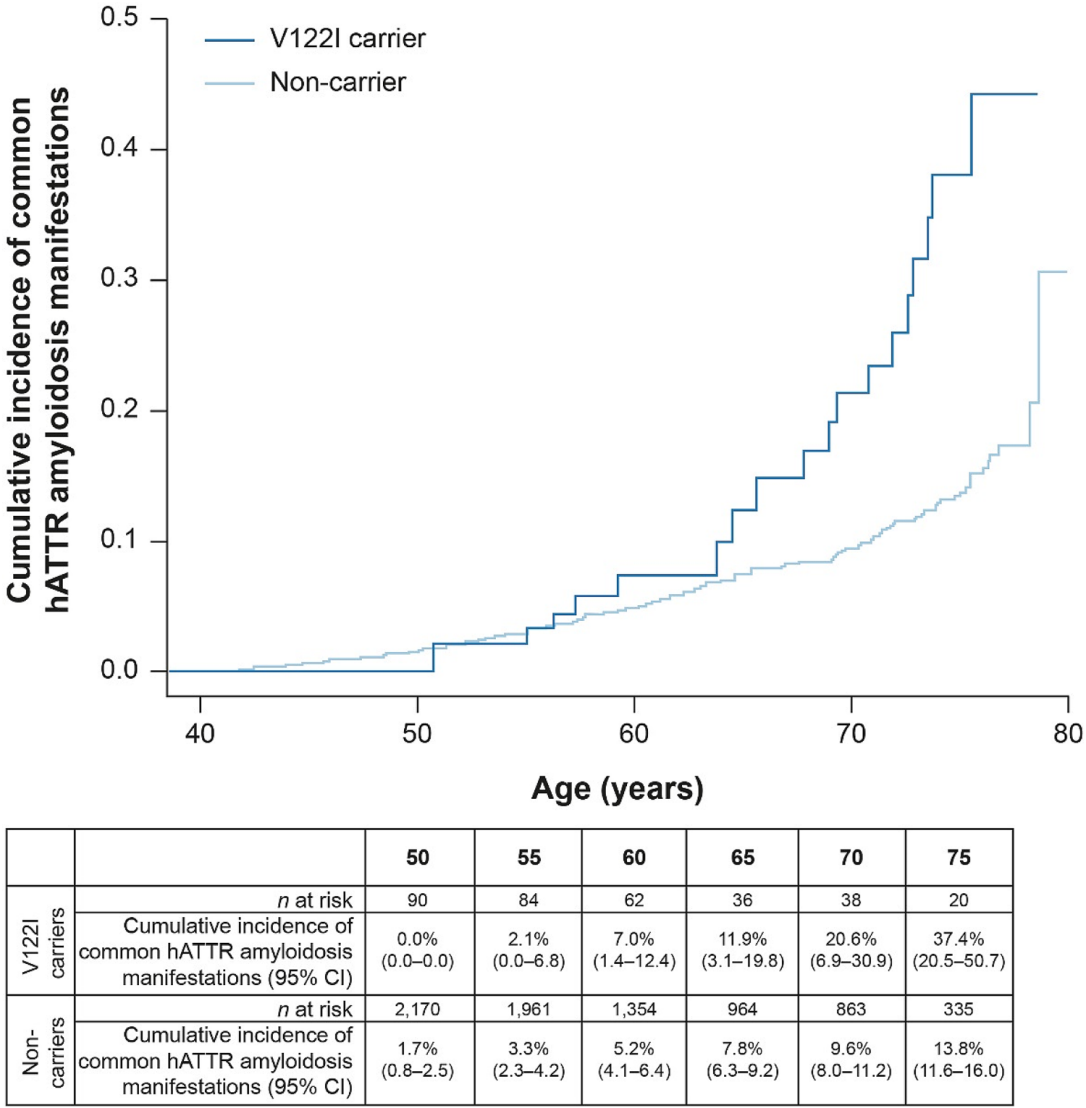
Cumulative incidence of common hATTR amyloidosis manifestations by V122I genotype in the UK Biobank (HR = 2.77, *p* = 2.62 × 10^–5^). *CI* confidence interval, *hATTR* hereditary transthyretin-mediated, *HR* hazard ratio, *V122I* valine-to-isoleucine substitution at position 122.

We tested the time to first diagnosis of a common hATTR amyloidosis manifestation by V122I genotype using Cox proportional hazards regression in the UK Biobank, and found a significant association (hazard ratio [HR] = 2.77, 95% CI 1.72–4.47, *p* = 2.62 × 10^–5^; Fig. 2). By age 65, the cumulative incidence of having at least one common hATTR amyloidosis manifestation among V122I carriers was 11.9% (95% CI 3.1–19.8), which increased by age 75 to 37.4% (95% CI 20.5–50.7) and was significantly higher than in non-carriers (13.8%, 95% CI 11.6–16.0).

**Figure 3:**
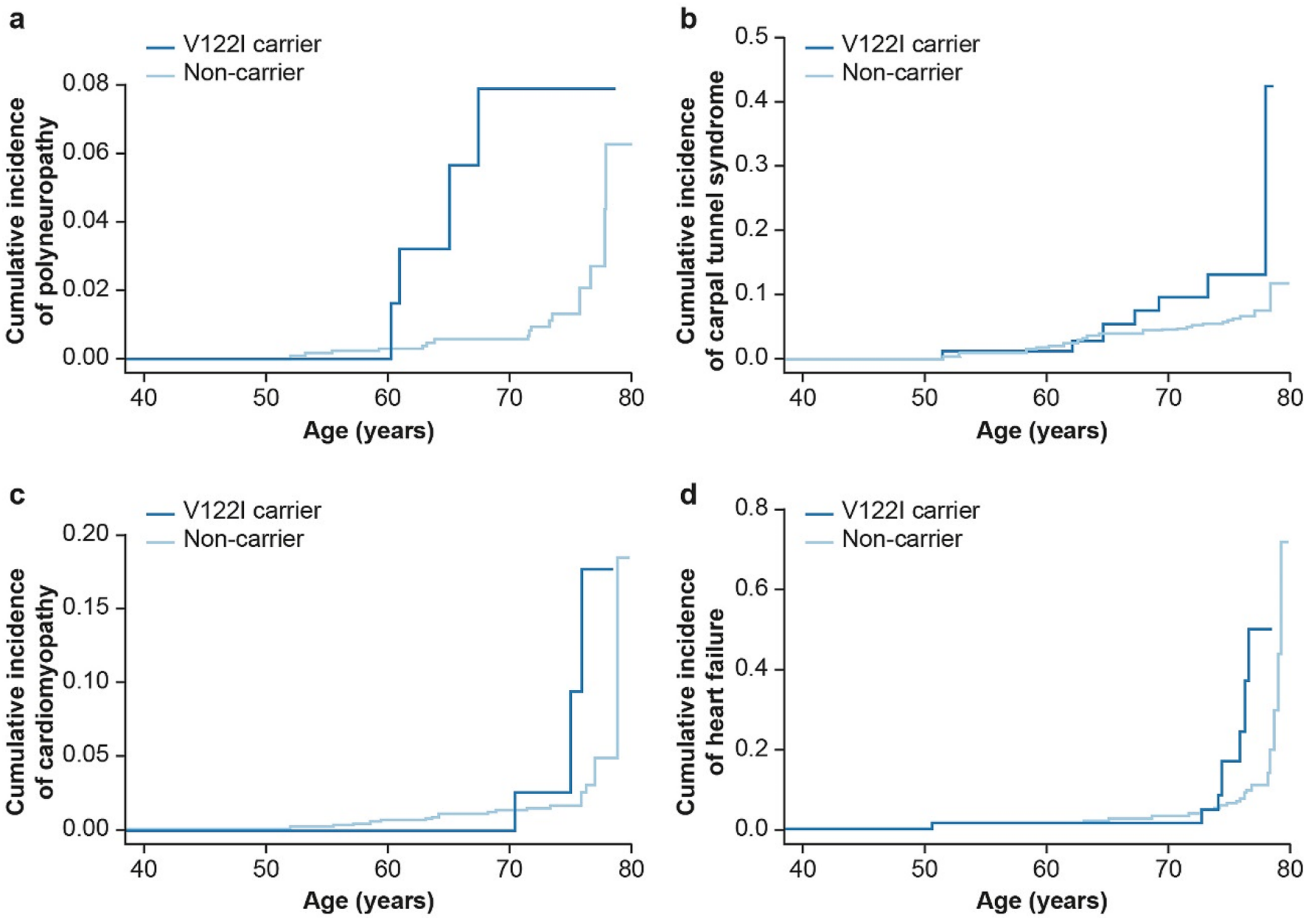
Cumulative incidence by V122I genotype of first diagnosis of common hereditary transthyretin-mediated amyloidosis manifestation: (**a**) polyneuropathy (G62), (**b**) carpal tunnel syndrome (G560), (**c**) cardiomyopathy (I42), and (**d**) heart failure (I50 or I098). *V122I* valine-to-isoleucine substitution at position 122.

The time to first common hATTR amyloidosis manifestation was assessed separately for each diagnosis (Fig. 3; Table S5 in the “Supplementary Information”), finding that V122I carriers had a significantly higher cumulative incidence of polyneuropathy, CTS, and HF than non-carriers (polyneuropathy HR = 6.9, *p* = 9.5 × 10^–4^; CTS HR = 2.7, *p* = 0.01; cardiomyopathy HR = 3.2, *p* = 0.06; HF HR = 3.2, *p* = 2.0 × 10^–3^). By age 75, 7.9% of V122I carriers had a polyneuropathy diagnosis, 13.2% had a CTS diagnosis, 2.5% had a cardiomyopathy diagnosis, and 16.7% had an HF diagnosis.

We performed the sex-stratified analysis of V122I with the composite phenotype of at least one common hATTR amyloidosis manifestation. A total of 130 women and 113 men were V122I carriers, including 10 men and 17 women with at least one common hATTR amyloidosis manifestation. A test of interaction by sex revealed no significant difference in time to first common hATTR amyloidosis manifestation by sex (*p* = 0.65). The V122I genotype was significantly associated with time to first hATTR amyloidosis manifestation in both men (HR = 3.3, *p* = 5.0 × 10^–4^) and women (HR = 2.5, *p* = 7.8 × 10^–3^) (see Fig. S3 in the “Supplementary Information”).

### Characteristics of V122I carriers with common hATTR amyloidosis manifestations in the UK Biobank

Logistic regression comparing V122I carriers with at least one common hATTR amyloidosis manifestation (*n* = 27) to those without any common manifestations (*n* = 216) revealed that those with manifestations were more likely to be older (*p* = 2.8 × 10^–6^) and current or former cigarette smokers (*p* = 1.4 × 10^–2^), although the association with smoking was not statistically significant when controlling for multiple comparisons. No sex difference was observed between those with and without any of the four common hATTR amyloidosis manifestations (*p* = 0.18) (Table 2).

**Table 2:** Characteristics of V122I carriers with ≥ 1 diagnosis of a common hATTR amyloidosis manifestation (polyneuropathy, carpal tunnel syndrome, cardiomyopathy, and heart failure).

	No common manifestations (*n* = 216)	At least one common manifestation (*n* = 27)	*p*
Mean (SD) age (years)	51.7 (7.75)	61.0 (7.80)	2.81 × 10^–6^
Male (%)	47.7	37.0	0.18
Mean (SD) BMI (kg/m^2^)	29.6 (5.03)	30.9 (5.88)	0.35
Smoking (%)	25.5	40.7	0.01
Genetic PC 1	384.0 (29.10)	384.0 (33.10)	0.89

### Population attributable risk of common hATTR amyloidosis manifestations to the V122I variant

To assess the impact of the V122I variant on common hATTR amyloidosis manifestations in the UK Biobank African ancestry population, we calculated the population attributable risk. Overall, 16.7% of the risk of a polyneuropathy diagnosis, 4.1% of the risk of a CTS diagnosis, 2.4% of the risk of a cardiomyopathy diagnosis, and 6.5% of the risk of an HF diagnosis were attributable to the V122I variant (Table 3).

**Table 3:** Attributable risk of common hATTR amyloidosis manifestations in the UK Biobank African ancestry population (*n* = 6062) due to the V122I variant.

	Diagnoses in V122I carriers (%) (*n*/*N*)	Population attributable risk to V122I variant (%)
Polyneuropathy	20.0 (5/25)	16.7
CTS	7.5 (13/174)	4.1
Cardiomyopathy	7.5 (3/40)	2.4
HF	10.2 (10/98)	6.5
At least one common hATTR amyloidosis manifestation	8.7 (27/311)	5.5

## Discussion

In this analysis of three large biobanks (UK Biobank, Penn Medicine Biobank, and Million Veteran Program) including 2739 carriers, the V122I variant was significantly associated with a polyneuropathy diagnosis. This suggests that individuals with the V122I genotype, who were historically assumed to be predominantly at risk for cardiomyopathy, have a significantly increased risk of polyneuropathy. In the UK Biobank, by age 75, 7.9% of V122I carriers had a clinical diagnosis of polyneuropathy. Moreover, V122I carriers represent one in five of all polyneuropathy diagnoses in this subpopulation of the UK Biobank. The V122I genotype has been linked to polyneuropathy in case reports and smaller studies^22–24^, as well as highlighted within a recent scientific statement from the American Heart Association^25^. However, to our knowledge, this is the first study to show a significant association between the V122I genotype and polyneuropathy. Detailed recommendations to improve the diagnosis of polyneuropathy in hATTR amyloidosis were recently published^26^.

Additionally, an overall enrichment of common hATTR amyloidosis manifestations (polyneuropathy, CTS, cardiomyopathy, and HF) was found among V122I carriers, including a statistically significant association between V122I and time to HF diagnosis (*p* = 2.0 × 10^–3^) and time to CTS diagnosis (*p* = 0.01). Despite this clear enrichment, only two out of 243 patients were diagnosed with amyloidosis (ICD10 “E85”), suggesting a considerable underdiagnosis of the disease consistent with previous literature^4,5^. Notably, the two participants with confirmed amyloidosis were diagnosed in their 70 s, with one dying from cardiac amyloid less than 2 years after diagnosis. This highlights the need for a better understanding of the early and multisystem manifestations of hATTR amyloidosis, particularly for V122I carriers, who have higher mortality rates and worse prognosis than patients with other cardiac pathologies^9,27–29^.

To identify the characteristics of participants with common hATTR amyloidosis manifestations, differences between V122I carriers with and without any diagnoses of the common hATTR amyloidosis manifestations were analyzed. V122I carriers with these diagnoses were older and more likely to be current/former smokers. We did not identify a difference between men and women in the prevalence of common hATTR amyloidosis manifestations, despite previous studies identifying gender as a significant modifier of disease penetrance^30^. While the two amyloidosis diagnoses were both in males, diagnoses of common hATTR amyloidosis manifestations were present in equal proportions of males and females.

The results are particularly important given recent advances in therapies for hATTR amyloidosis. Two major strategies have been employed: (1) reduce serum TTR levels by inhibiting hepatic synthesis of TTR proteins through RNA interference therapeutics (patisiran) or antisense oligonucleotides (inotersen); and (2) prevent dissociation of the TTR tetramer through small molecule TTR stabilizers (tafamidis). Both approaches have yielded positive results in phase III studies of hATTR amyloidosis with polyneuropathy^31,32^, and tafamidis has also shown benefit in patients with ATTR amyloidosis with cardiomyopathy^33^, with studies underway for patisiran^34^ and inotersen^35^. For patients with hATTR amyloidosis to derive maximal benefit from the available therapies, early identification of patients remains essential.

Previous studies of V122I carriers have captured patients late in their disease course and/or looked at a limited set of predefined outcomes^36^. The main strength of this study is its size and prospective nature. Moreover, we identify treatable clinical manifestations associated with carrying a variant common in individuals of African ancestry, who are historically understudied in genetic research^37,38^. However, this study must be interpreted in the context of potential limitations. Analyses in the UK Biobank were performed using inpatient hospital ICD10 diagnosis codes and do not capture outpatient or self-reported diagnoses. This is especially important to note as diagnosing polyneuropathy can be highly dependent on provider specialty as well as the setting in which the patient is seen (i.e., inpatient versus outpatient). We did not have complete records of medical provider specialty and therefore could not evaluate or control for this in our analyses. It is possible our analyses missed additional hATTR amyloidosis manifestations present in participants but not captured in the hospital ICD10 diagnosis codes. Effect estimates of the association between the V122I variant and polyneuropathy were heterogeneous between the three biobanks (OR UK Biobank = 6.4; OR Penn Medicine Biobank = 1.6; OR Million Veteran Program = 1.5). This is likely due to differences in the collection methods of ICD diagnosis codes: in the UK Biobank, diagnoses were from inpatient hospital records, whereas in the Penn Medicine Biobank and the Million Veteran Program they included both inpatient and outpatient diagnoses. Indeed, the rate of polyneuropathy in non-V122I carriers is lower in the UK Biobank (0.3%) than non-carriers in the Penn Medicine Biobank (5.0%) or the Million Veteran Program (3.3%), suggesting that the higher OR in this population may be due to the method of ICD10 diagnosis collection.

Additional limitations include that UK Biobank participants are healthier on average than the general UK population^39^. Also, despite the large sample size, the number of V122I carriers with common hATTR amyloidosis manifestations in the UK Biobank is small (*n* = 27), limiting statistical power to detect significant associations. Furthermore, V122I carriers in the UK Biobank were on average 60 years of age at censoring (range: 42–79 years), which is younger than the age range during which V122I hATTR amyloidosis typically presents^36^. We hypothesize that this younger age may be a reason that the well-known cardiac manifestations of hATTR amyloidosis are not as enriched as expected based on previous reports and our study may underrepresent the true impact of the V122I variant. Lastly, while four common hATTR amyloidosis manifestations were found to be enriched in V122I carriers, not all diagnoses of cardiomyopathy, CTS, HF, and polyneuropathy were confirmed to be due to hATTR amyloidosis.

In a large study of one of the most common pathogenic *TTR* mutations, the V122I variant was significantly associated with polyneuropathy diagnosis. This finding has important clinical implications, as physicians should have a high clinical suspicion for the multisystem manifestations of hATTR amyloidosis in V122I carriers, including both cardiomyopathy and polyneuropathy.

## Supplementary Information

Supplementary Information.

## Data Availability

UK Biobank data are available upon application to the UK Biobank. Data from the Penn Medicine Biobank and Million Veteran Program will be made available for purposes of replication of the findings upon reasonable request and with appropriate Institutional Review Board approval and materials transfer agreement.
