# Supplementary material for: Association of the transthyretin variant V122I with polyneuropathy among individuals of African ancestry
Source: Sci Rep. 2021 Jun 2;11:11645. doi: 10.1038/s41598-021-91113-6 (PMC8172853; doi:10.1038/s41598-021-91113-6)
Supplement: Supplementary file 1 — Supplementary Information. [file 41598_2021_91113_MOESM1_ESM.docx]

**Association of the transthyretin variant V122I with polyneuropathy among individuals of African ancestry**

Margaret M. Parker^1^, Scott M. Damrauer^2,3^, Catherine Tcheandjieu^4,5^, David Erbe^1^, Emre Aldinc^1^, Philip N. Hawkins^6^, Julian D. Gillmore^6^, Leland E. Hull^7,8^, Julie A. Lynch^9,10^, Jacob Joseph^11,12^, Simina Ticau^1^, Alexander O. Flynn-Carroll^1^, Aimee M. Deaton^1^, Lucas D. Ward^1^, Themistocles L. Assimes^4,5^, Philip S. Tsao^4,5^, Kyong-Mi Chang^3,13^, Daniel J. Rader^13,14^, Kevin Fitzgerald^1^, Akshay K. Vaishnaw^1^, Gregory Hinkle^1^ & Paul Nioi^1^*

^1^Alnylam Pharmaceuticals, Cambridge MA 02142, USA. ^2^Department of Surgery, Perelman School of Medicine, University of Pennsylvania, Philadelphia, PA 19104, USA. ^3^The Corporal Michael J. Crescenz VA Medical Center, Philadelphia, PA 19104, USA. ^4^12 VA Palo Alto Health Care System, Palo Alto, CA 94304, USA. ^5^Department of Medicine, Stanford University School of Medicine, Stanford, CA 94304, USA. ^6^Centre for Amyloidosis & Acute Phase Proteins, Division of Medicine UCL (Royal Free Campus), London NW3 2PF, UK. ^7^Division of General Internal Medicine, Massachusetts General Hospital, Boston, MA 02114, USA. ^8^Center for Healthcare Organization and Implementation Research, Edith Nourse Rogers Memorial Veterans Hospital, Bedford, MA 01730, USA. ^9^School of Nursing & Health Sciences, University of Massachusetts, Boston, MA 02125, USA. ^10^VA Informatics and Computing Infrastructure (VINCI), VA Salt Lake City Health Care System, Salt Lake City, UT 84148, USA. ^11^Department of Medicine, Veterans Affairs Boston Healthcare System, Boston, MA 02130, USA. ^12^Department of Medicine, Brigham and Women’s Hospital and Harvard Medical School, Boston, MA 02115, USA. ^13^Department of Medicine, Perelman School of Medicine, University of Pennsylvania, Philadelphia, PA 19104, USA. ^14^Department of Genetics, Perelman School of Medicine, University of Pennsylvania, Philadelphia, PA 19104, USA.

**Correspondence:**
Paul Nioi
[pnioi@alnylam.com](mailto:pnioi@alnylam.com)

# Supplementary information

# Materials and methods

**Study populations.** *UK Biobank*. The UK Biobank is a large, population-based, prospective cohort study which recruited 502,634 participants aged 40–69 years in England, Wales, and Scotland between 2006 and 2010^1^. Participants in the UK Biobank lived within 25 miles of one of the 22 assessment centers and participated in a baseline assessment involving the collection of extensive data from questionnaires, health records, physical measurements, imaging, and biologic samples^1^. The UK Biobank recruited a population-based sample without selection on any disease outcome, but subjects were generally healthier than the overall National Health Service (NHS) population at baseline^2^. Data used in this analysis were accessed through UK Biobank application no. 26,041 and are publicly available upon request to the UK Biobank. A total of 494,078 samples (including duplicates) were sent to Affymetrix for genotyping, with results from 488,377 included in the released data following quality control analysis^3^.

All UK Biobank study participants underwent a baseline study assessment where covariates including age, sex, body mass index, self-reported diabetes diagnosis, self-reported hypertension diagnosis, and smoking status were collected. Additionally, participants consented to access to their NHS hospital records, which were used to obtain primary and secondary International Statistical Classification of Diseases and Related Health Problems, 10th Revision (ICD10) disease diagnosis codes. Diagnosis codes were collected prospectively from inpatient NHS visits during study follow-up and retrospectively from 1996 to baseline visit.

*Penn Medicine Biobank.* The Penn Medicine Biobank currently includes over 60,000 participants enrolled through the University of Pennsylvania Health System who have consented to allow the linkage of biospecimens to their longitudinal electronic health record (EHR). The current analysis utilizes data on 5,737 individuals of genetically inferred African ancestry recruited between November 21, 2008, and January 4, 2017, that passed quality control as described in detail elsewhere^4^.

*Million Veteran Program*. The Million Veteran Program is a large, multiethnic cohort within the U.S. Department of Veterans Affairs (VA). More than 825,000 veterans over age 18 years were recruited from 63 participating VA medical center facilities across the United States between 2011 and 2017^5^. The Million Veteran Program biobank incorporates data from biospecimens, baseline and lifestyle surveys, and EHRs, including clinical laboratory measurements, diagnostic imaging reports, diagnosis and procedure codes, and vital status. Information from Medicare claims is not included in the EHR data. Million Veteran Program research protocols were approved by the VA Central Institutional Review Board. All participants provided informed consent and authorization for review of their medical records.

**Phenome-wide association analysis and replication.** Phenome-wide association analysis was performed in PLINK (v2.0)^6^ using logistic regression with the “firth-fallback” option, which runs Firth regression^7^ when logistic regression fails due to a rare outcome. Analyses were performed using the REVEAL/SciDB translational analytics platform from Paradigm4. The significant association between the valine-to-isoleucine substitution at position 122 (V122I) variant and polyneuropathy diagnosis was replicated in the Penn Medicine Biobank and the Million Veteran Program. In the Penn Medicine Biobank, polyneuropathy was defined as the assignment of the ICD9 diagnosis code 357 or ICD10 diagnosis codes of “G62” or “G63” on two or more separate dates. Association was assessed using logistic regression, controlling for age, sex, and the first five principal components. In the Million Veteran Program, polyneuropathy was defined as a diagnosis of “G62.” Association was assessed using logistic regression, controlling for age, sex, and the first 10 principal components of genetic ancestry in 82,362 unrelated Million Veteran Program participants of African ancestry. For the sensitivity analyses including diabetes as a covariate, type 2 diabetes was defined as: 1) a diagnosis of the ICD10 code “E11” in the UK Biobank; 2) using a combination of ICD9 and ICD10 codes, laboratory tests, and diabetes medication use in the Penn Medicine Biobank; and 3) Phecodes 250.2, 250.21, 250.22, 250.23, 250.24, and 250.25 made from aggregated ICD10 codes in the Million Veteran Program.

**Assessment of common hATTR amyloidosis manifestations in V122I carriers.** Time to first diagnosis of common hereditary transthyretin-mediated (hATTR) amyloidosis manifestations (polyneuropathy [“G62”], carpal tunnel syndrome [“G560”], cardiomyopathy [“I42”], or heart failure [“I50” or “I098”]) was tested in the UK Biobank unrelated African ancestry population. For time to event analyses, prevalent diagnoses occurring before a patient’s date of enrollment were removed. Time on study was calculated as date of enrollment to date of first primary or secondary diagnosis of an hATTR amyloidosis manifestation (if participant had a diagnosis), date of loss to follow-up (if participant was lost to follow-up), or date of administrative censoring (March 31, 2017 for participants in England, February 29, 2016 for participants in Wales, or October 31, 2016 for participants in Scotland). Age at diagnosis was calculated from month and year of birth. Data availability dates were obtained from: <http://biobank.ctsu.ox.ac.uk/crystal/exinfo.cgi?src=Data_providers_and_dates> (accessed on April 1, 2019). Survival analyses were performed in R version 3.4.4 using the survival (v. 2.43) and survminer packages (v. 0.4.3). All analyses were controlled for known confounders including age, sex, smoking status, and genetic ancestry via 10 genetic principal components.

**Population attributable risk to the V122I variant.** The population attributable risk of common hATTR amyloidosis manifestations due to the V122I variant was calculated in the unrelated African ancestry subpopulation of the UK Biobank (*n =* 6,062). The following equation was used, where risk was estimated using the cumulative incidence of disease at age 75 from the Kaplan–Meier curve:

$$Population attributable risk= \frac{N V122I with disease}{N total with disease} \times\frac{CI V122I-CI non-carrier}{CI V122I}$$

CI, cumulative incidence.

# References

1. Allen, N. *et al*. UK Biobank: current status and what it means for epidemiology*.* *Health Policy Technol.* **1**(3), 123–126 (2012).
2. Fry, A. *et al*. Comparison of sociodemographic and health-related characteristics of UK Biobank participants with those of the general population. *Am. J. Epidemiol.* **186**(9), 1026–1034 (2017).
3. Bycroft, C. *et al*. The UK Biobank resource with deep phenotyping and genomic data*.* *Nature* **562**(7726), 203–209 (2018).
4. Damrauer, S. M. *et al*. Association of the V122I hereditary transthyretin amyloidosis genetic variant with heart failure among individuals of African or Hispanic/Latino ancestry*.* *JAMA.* **322**(22), 2191–2202 (2019).
5. Gaziano, J. M. *et al*. Million Veteran Program: a mega-biobank to study genetic influences on health and disease*.* *J. Clin. Epidemiol.* **70**, 214–223 (2016).
6. Chang, C. C. *et al*. Second-generation PLINK: rising to the challenge of larger and richer datasets*.* *Gigascience* **4**, 7 (2015).
7. Wang, X. Firth logistic regression for rare variant association tests. *Front Genet.* **5**,187 (2014).


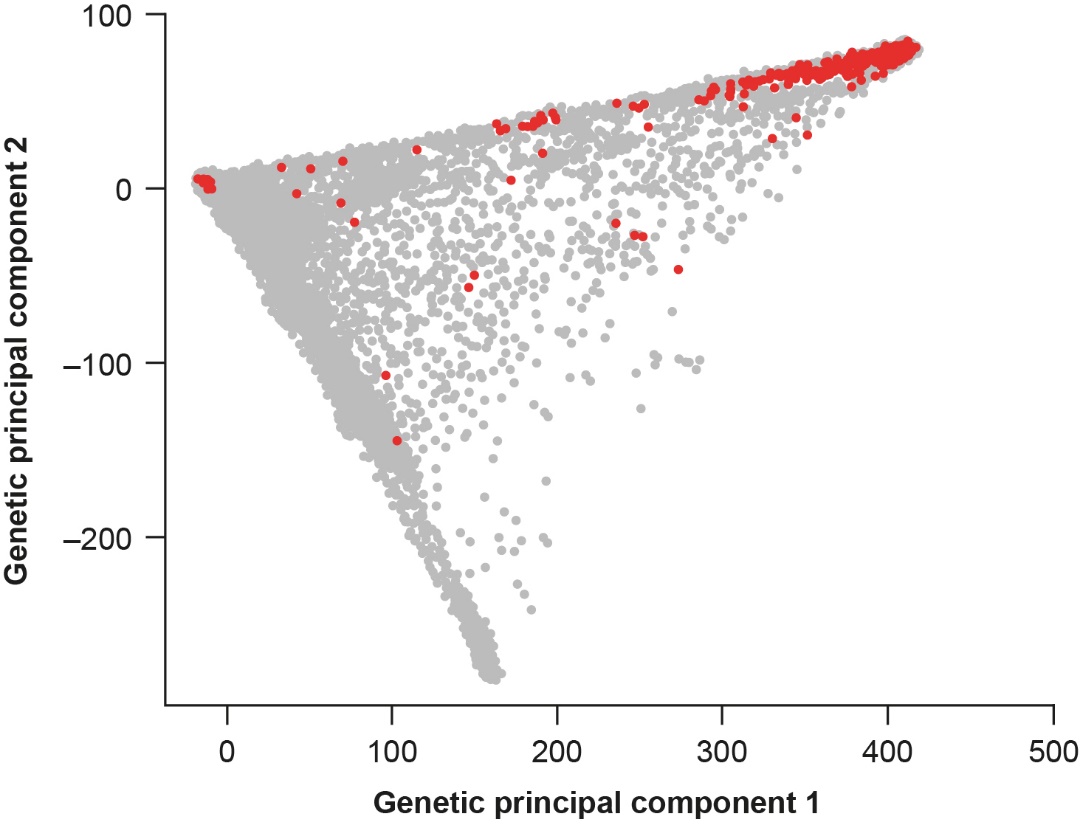


**Supplementary Figure S1.** Genetic principal component 1 versus genetic principal component 2 with V122I carriers highlighted in red (*n* = 387 V122I carriers). The majority (80.6%, *n* = 327) of V122I carriers are of self-reported African ancestry. After removing population outliers based on genetic principal components and related individuals (first- and second-degree relatives), the MAF of V122I in the four UK Biobank subpopulations was: 1) MAF African ancestry subpopulation = 0.02 (*n* = 243/6,062); 2) MAF European ancestry subpopulation = 1.78 × 10^–5^ (*n* = 12/337,126); 3) MAF Southeast Asian ancestry subpopulation = 0.0 (*n* = 0/7,453); 4) MAF Chinese ancestry subpopulation = 0.0 (*n* = 0/1,340). MAF, minor allele frequency; V122I, valine-to-isoleucine substitution at position 122.


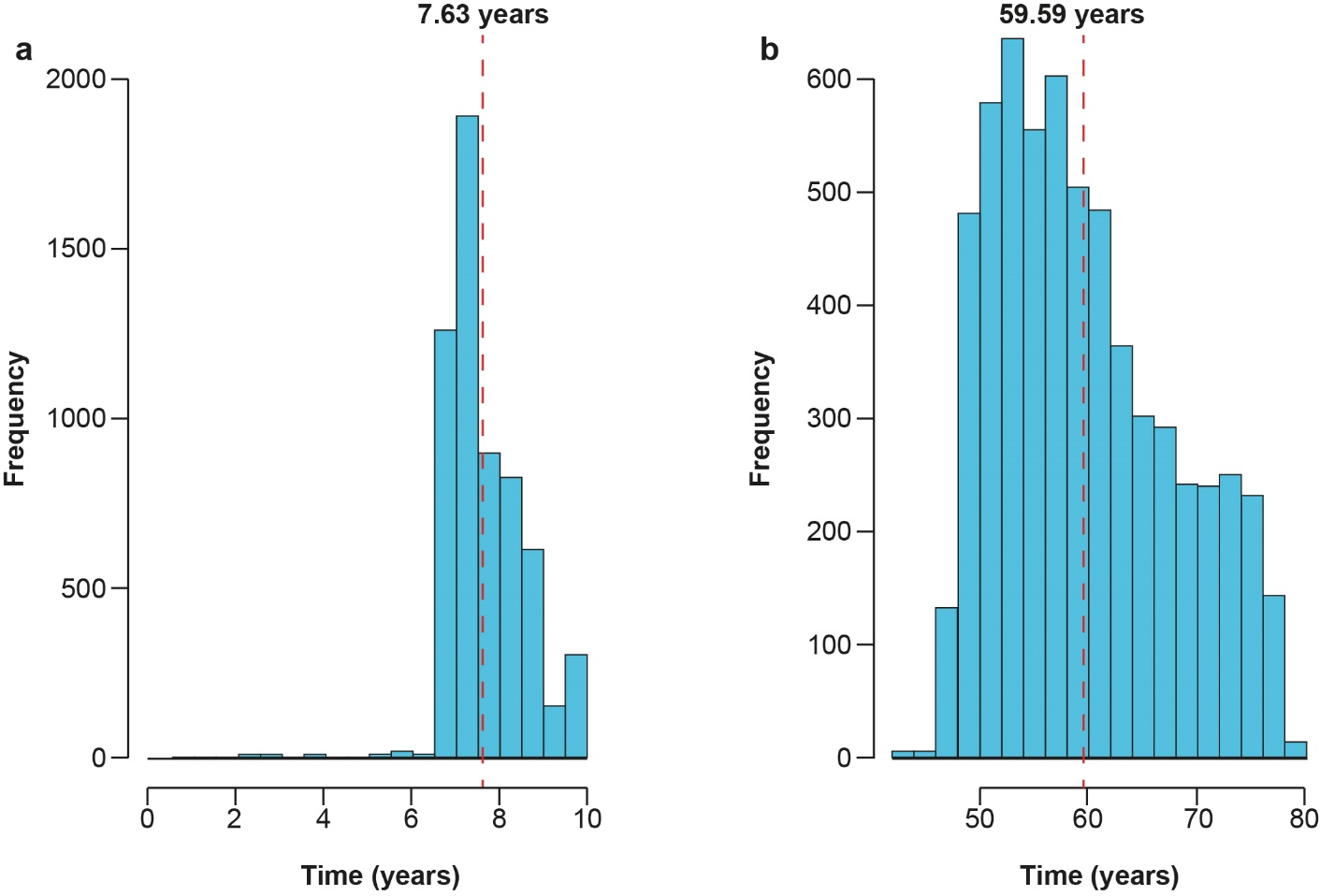


**Supplementary Figure S2.** Histograms of (**a**) follow-up time in unrelated African ancestry participants of the UK Biobank study (mean: 7.6 years, range: 0.22–10.0 years) and (**b**) age at last known UK Biobank observation (mean: 59.6 years, range: 42.2–79.0 years). Censoring occurred at date of death (if died), date of loss to follow-up (if lost to follow-up), or March 31, 2017, for participants in England, February 29, 2016, for participants in Wales, or October 31, 2016 for participants in Scotland. UK Biobank participants in this study were followed for a total of 46,260.8 years of person-time.


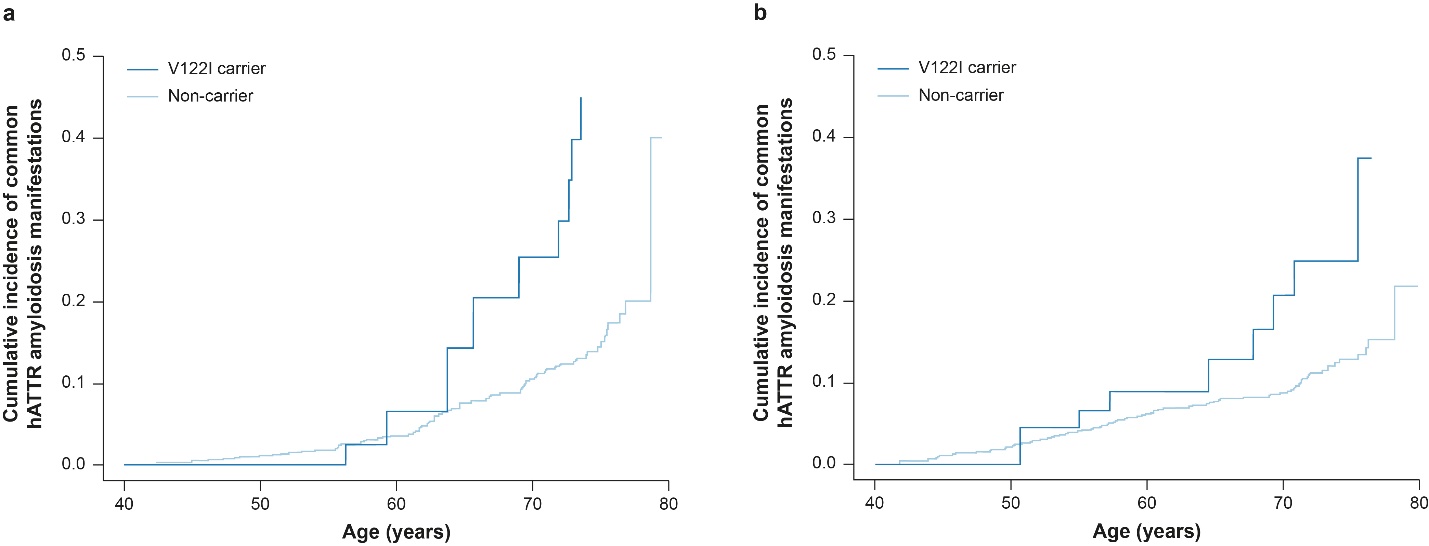


**Supplementary Figure S3.** Sex-stratified cumulative incidence of common hATTR amyloidosis manifestations by V122I genotype in the UK Biobank (**a**) men and (**b**) women. hATTR, hereditary transthyretin-mediated; V122I, valine-to-isoleucine substitution at position 122.

| **Phenotypes** | **Non-carriers** | **Carriers** | ***p*** |
| --- | --- | --- | --- |
| *n* | 5,547 | 190 |  |
| Age, mean (SD) | 55 (16) | 53 (17) | 0.18 |
| Male, *n* (%) | 2,172 (39%) | 83 (44%) | 0.23 |
| BMI, mean (SD) | 32 (8.2) | 32 (8.4) | 0.53 |
| Hypertension, *n* (%) | 3,444 (62%) | 116 (61%) | 0.83 |
| Diabetes, *n* (%) | 1,577 (28%) | 60 (32%) | 0.39 |
| Current smoker, *n* (%) | 772 (14%) | 18 (9.4%) | 0.10 |

**Supplementary Table S1.** Baseline characteristics of the African ancestry Penn Medicine study population by V122I genotype.

| **Phenotypes** | **Non-carriers** | **Carriers** | ***p*** |
| --- | --- | --- | --- |
| *n* | 80,031 | 2,305 |  |
| Age, mean (SD) | 57.6 (12.2) | 57.3 (12.0) | 0.14 |
| Male, *n* (%) | 69,294 (86.6%) | 1,974 (85.6%) | 0.12 |
| BMI, mean (SD) | 35.9 (8.2) | 35.8 (8.0) | 0.58 |
| Hypertension, *n* (%) | 28,137 (35.2%) | 740 (32.1%) | 0.02 |
| Diabetes, *n* (%) | 12,308 (15.4%) | 327 (14.2%) | 0.46 |
| Current smoker, *n* (%) | 10,909 (13.6%) | 303 (13.2%) | 0.34 |

**Supplementary Table S2.** Characteristics of V122I carriers and non-carriers in the Million Veteran Program. Characteristics were assessed at enrollment. Only a subset of participants had data on hypertension, diabetes, and smoking status.

| **ICD10 diagnosis code** | **OR (95% CI)** | **SE** | ***p*** |
| --- | --- | --- | --- |
| Polyneuropathy (G62) | 6.40 (2.63–15.55) | 0.45 | 4.24 × 10^–5^ |
| Other disorders of synovium and tendon (M67) | 3.22 (1.66–6.26) | 0.34 | 5.40 × 10^–4^ |
| Unspecified lump in breast (N63) | 5.29 (2.00–13.98) | 0.50 | 7.80 × 10^–4^ |
| Epididymitis (N45) | 6.54 (2.17–19.76) | 0.56 | 8.66 × 10^–4^ |
| Heart failure (I50) | 2.50 (1.33–4.68) | 0.32 | 4.35 × 10^–3^ |
| Unspecified right bundle-branch block (I45) | 3.67 (1.46–9.21) | 0.47 | 5.60 × 10^–3^ |
| Retention of urine (R33) | 2.52 (1.30–4.88) | 0.34 | 6.36 × 10^–3^ |
| Mononeuropathies of upper limb (G56) | 1.94 (1.11–3.39) | 0.29 | 1.98 × 10^–2^ |
| Postprocedural disorders of digestive system (K91) | 4.20 (1.25–14.13) | 0.62 | 2.03 × 10^–2^ |
| Overexertion and strenuous or repetitive movements (X50) | 3.82 (1.14–12.75) | 0.62 | 2.95 × 10^–2^ |
| Non-rheumatic mitral value insufficiency (I34) | 3.00 (1.11–8.14) | 0.51 | 3.09 × 10^–2^ |
| Other enthesopathies (M77) | 3.53 (1.08–11.54) | 0.60 | 3.68 × 10^–2^ |
| Ascites (R18) | 2.93 (1.05–8.18) | 0.52 | 3.99 × 10^–2^ |
| Other dermatitis (L30) | 3.33 (1.02–10.85) | 0.60 | 4.63 × 10^–2^ |
| Umbilical hernia (K42) | 2.04 (0.99–4.18) | 0.37 | 5.17 × 10^–2^ |

**Supplementary Table S3.** Top results from the phenome-wide association analysis of V122I variant and ICD10 diagnosis codes in the unrelated African ancestry population of the UK Biobank.

|  | Cases | Controls | OR | 95% CI | *p* |
| --- | --- | --- | --- | --- | --- |
| Amyloidosis | 869 | 81,513 | 1.90 | 1.38–2.62 | 9.49 × 10^−5^ |
| Cardiomyopathy | 3,967 | 78,415 | 1.34 | 1.12–1.61 | 1.42 × 10^−3^ |
| Carpal tunnel syndrome | 4,711 | 77,671 | 1.69 | 1.45–1.97 | 1.95 × 10^−11^ |
| Heart failure | 8,717 | 73,665 | 1.32 | 1.16–1.51 | 2.82 × 10^−5^ |
| Polyneuropathy | 2,745 | 79,637 | 1.48 | 1.21–1.82 | 1.79 × 10^−4^ |

**Supplementary Table S4.** Association of the V122I variant with hATTR amyloidosis diagnosis and common hATTR amyloidosis manifestations in the Million Veteran Program cohort (*n* = 2,306 V122I carriers and 80,076 non-carriers). Results are from a logistic regression controlling for age, sex, and genetic ancestry via the first 10 principal components.

| **Age** | **Cumulative incidence  V122I carriers (95% CI)** | **Cumulative incidence  non-carriers (95% CI)** |
| --- | --- | --- |
| Polyneuropathy | | |
| 50 | 0.0% (0.0–0.0) | 0.0% (0.0–0.0) |
| 55 | 0.0% (0.0–0.0) | 0.2% (0.0–0.4) |
| 60 | 0.0% (0.0–0.0) | 0.3% (0.1–0.5) |
| 65 | 5.6% (0.0–11.8) | 0.6% (0.2–0.9) |
| 70 | 7.9% (0.0–15.2) | 0.6% (0.2–0.9) |
| 75 | 7.9% (0.0–15.2) | 1.3% (0.5–2.1) |
| Carpal tunnel syndrome | | |
| 50 | 0.0% (0.0–0.0) | 0.2% (0.0–0.3) |
| 55 | 1.2% (0.0–3.4) | 1.0% (0.6–1.4) |
| 60 | 1.2% (0.0–3.4) | 2.0% (1.4–2.6) |
| 65 | 5.4% (0.0–11.4) | 3.9% (3.0–4.9) |
| 70 | 9.9% (1.0–17.9) | 4.6% (3.5–5.7) |
| 75 | 13.2% (2.2–23.0) | 6.0% (4.6–7.5) |
| Cardiomyopathy | | |
| 50 | 0.0% (0.0–0.0) | 0.0% (0.0–0.0) |
| 55 | 0.0% (0.0–0.0) | 0.2% (0.0–0.4) |
| 60 | 0.0% (0.0–0.0) | 0.7% (0.3–1.0) |
| 65 | 0.0% (0.0–0.0) | 1.1% (0.6–1.7) |
| 70 | 0.0% (0.0–0.0) | 1.3% (0.7–2.0) |
| 75 | 2.5% (0.0–7.2) | 1.7% (0.9–2.4) |
| Heart failure | | |
| 50 | 0.0% (0.0–0.0) | 0.2% (0.0–0.3) |
| 55 | 1.1% (0.0–3.1) | 1.0% (0.6–1.4) |
| 60 | 1.1% (0.0–3.1) | 2.0% (1.4–2.6) |
| 65 | 1.1% (0.0–3.1) | 3.9% (3.0–4.9) |
| 70 | 1.1% (0.0–3.1) | 4.6% (3.5–5.7) |
| 75 | 16.7% (1.1–29.8) | 6.0% (4.6–7.5) |

**Supplementary Table 5.** Cumulative incidence of common hATTR amyloidosis manifestations by age. Estimates from Kaplan–Meier analysis testing time to first common hATTR amyloidosis manifestation by V122I genotype during UK Biobank study follow-up. Prevalent diagnoses occurring before a patient’s date of enrollment were removed. The association of V122I genotype with time to first common manifestation is tested using Cox proportional hazards regression controlling for age, sex, smoking, and genetic ancestry via 10 genetic principal components (polyneuropathy HR = 6.9, 95% CI = 2.2–21.7, *p* = 9.5 × 10^–4^; carpal tunnel syndrome HR = 2.7, 95% CI = 1.2–5.8, *p*= 0.01; cardiomyopathy HR = 3.2, 95% CI = 0.9–10.7, *p* = 0.06; heart failure HR = 3.2, 95% CI = 1.5–6.9, *p* = 0.002).
